# Highly Polarized Single-Photon Emission from Localized Excitons in a WSe_2_/CrSBr Heterostructure

**DOI:** 10.1021/acsphotonics.5c00144

**Published:** 2025-05-29

**Authors:** Varghese Alapatt, Francisco Marques-Moros, Carla Boix-Constant, Samuel Mañas-Valero, Kirill I. Bolotin, Josep Canet-Ferrer, Eugenio Coronado

**Affiliations:** † Instituto de Ciencia Molecular (ICMol), Universitat de València, c/Catedrático José Beltrán 2, 46980 Paterna, Spain; ‡ Kavli Institute of Nanoscience, Delft University of Technology (TU Delft), Lorentzweg 1, 2628 CJ Delft, The Netherlands; § Department of Physics, 240407 Freie Universität Berlin, Arnimallee 14, 14195 Berlin, Germany

**Keywords:** transition metal dichalcogenides, magnetic proximity effects, 2D magnets, van der Waals heterostructures, quantum emitters, magneto-optics

## Abstract

Single-photon emitters (SPEs) are crucial in quantum communication and information processing. In 2D transition metal dichalcogenides (TMDs), SPEs are realized through inhomogeneous strain, while in combination with 2D magnets, a high spontaneous out-of-plane magnetization can be induced due to proximity effects. Here, an alternative is proposed that consists of suspending a TMD monolayer (WSe_2_) on a few-layer antiferromagnet (CrSBr) with in-plane magnetic ordering. The resulting heterostructure exhibits localization centers at lower energies than expected. Among them, a bright SPE with a high degree of polarization selection is identified. This suffers a clear energy shift driven by an in-plane magnetic field, and interestingly, this shift is correlated with the metamagnetic transition of CrSBr, suggesting a new kind of proximity-type effect. Unlike regular SPEs in WSe_2_ (sensitive to out-of-plane magnetic fields), our SPE demonstrates sensitivity to both in-plane and out-of-plane magnetic fields. The added tunability at significantly lower fields offers a promising direction for developing magnetically responsive quantum emitters, paving the way for more practical applications in quantum technologies.

## Graphical abstract

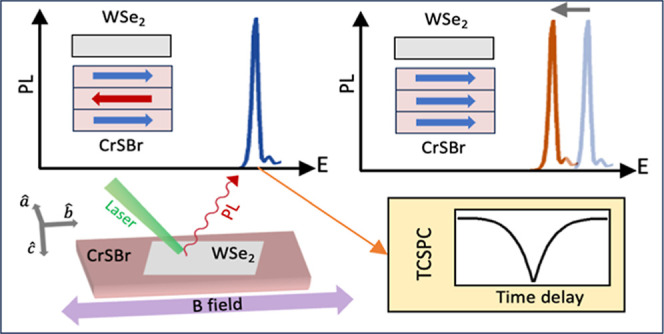

## Introduction

Single-photon emission is a fundamental constituent in quantum information technologies^1,2^ and quantum photonics.^3,4^ Atomic defects^5^ and localized strain in monolayer WSe_2_ have been extensively reported to facilitate the localization of excitons that exhibit quantum emitter properties.^6–9^ Besides being easy to integrate with other nanostructures,^10–12^ monolayers of WSe_2_ also afford an additional valley degree of freedom due to the presence of inequivalent K valleys in their electronic structure.^13,14^ The flexibility of this 2D material enables the engineering of a local strain-induced potential. Such an approach is the most successful in generating single-photon emitters (SPEs) in 2D systems. However, crucial requirements for the SPEs to be integrated into photonic devices are the efficiency of the quantum emission and tunable polarization.^1,15,16^ Despite the progress made in the field of strain engineering,^17^ polarization of the strain-induced SPEs is dependent largely on the nature and orientation of the strain tensor,^17,18^ in addition to the electron–hole spin-exchange interaction.^6–8,19^

Further functionalities have been induced through the integration of transition metal dichalcogenides (TMDs) with other 2D materials.^20–24^ The resulting heterostructures can modify or enhance the optical response to electric fields,^25^ magnetic fields,^24,26^ or mechanical strain^27^ of the semiconductor constituent. For example, 2D ferromagnets like CrI_3_ ^28–30^ can induce spontaneous valley splitting and enhanced valley polarization by out-of-plane magnetic fields.^28,31,32^ In addition, the presence of spin-selective magnetic centers (e.g., Cr^3+^ in materials such as CrI_3_ ^26,28^ or CrBr_3_ ^33,34^ ) enables further out-of-plane magnetic field dependence.

As an alternative, in this work, we explore the use of the 2D A-type antiferromagnet CrSBr^35–37^ in combination with WSe_2_. This combination opens the possibility of investigating proximity effects due to the in-plane magnetic ordering of CrSBr.^24,38–40^ Among the different kinds of localization centers observed in this heterostructure, we found a highly polarized SPE without any measurable zero-field splitting and controllable by means of an in-plane magnetic field. In particular, the emission of this SPE is red-shifted, coinciding with the flip-field from the antiparallel- to the parallel-spin configuration, around 200 mT. This exotic combination of properties enables applications requiring significantly lower field strength than conventional out-of-plane fields. Importantly, the lack of observable zero-field splitting together with the less demanding field strengths will enable quicker magnetic switching.

## Results

Our heterostructure is composed of a monolayer of WSe_2_ on top of a six-layer CrSBr, held together by van der Waals interactions (Figure 1a). The crystal structures of both constituents are depicted in Figure 1b,c. Figure 1d shows a set of PL color maps of the sample, where the color bar represents the integrated intensity of the microphotoluminescence (μ-PL). In the top and bottom panels of Figure 1d the position of the CrSBr (1.3–1.38 eV) and WSe_2_ (1.55–1.75 eV) flakes can be distinguished by filtering their corresponding emission energies. The integrated intensity over the whole spectra is shown in the middle panel. Figure 1e shows reference μ-PL spectra from CrSBr and WSe_2_, top and bottom panels, respectively. The reference signal from CrSBr is acquired from the same flake forming the heterostructure (far from the overlap with WSe_2_), while the reference signal from WSe_2_ is acquired from the triangular flake close to the heterostructure. A representative spectrum of the WSe_2_/CrSBr heterostructure is shown in the middle panel. All these spectra have been collected at 30 K, because, at this temperature, the neutral (X^0^ ∼ 1.73 eV) and charged exciton (X^–^ ∼ 1.7 eV) are clearly identified.^41,42^ The evolution of the PL with temperature is shown in Figure S4. The broad emission band of WSe_2_ around 1.6 eV energy is typically attributed to localized excitons (LEs).^42,43^

**Figure 1:**
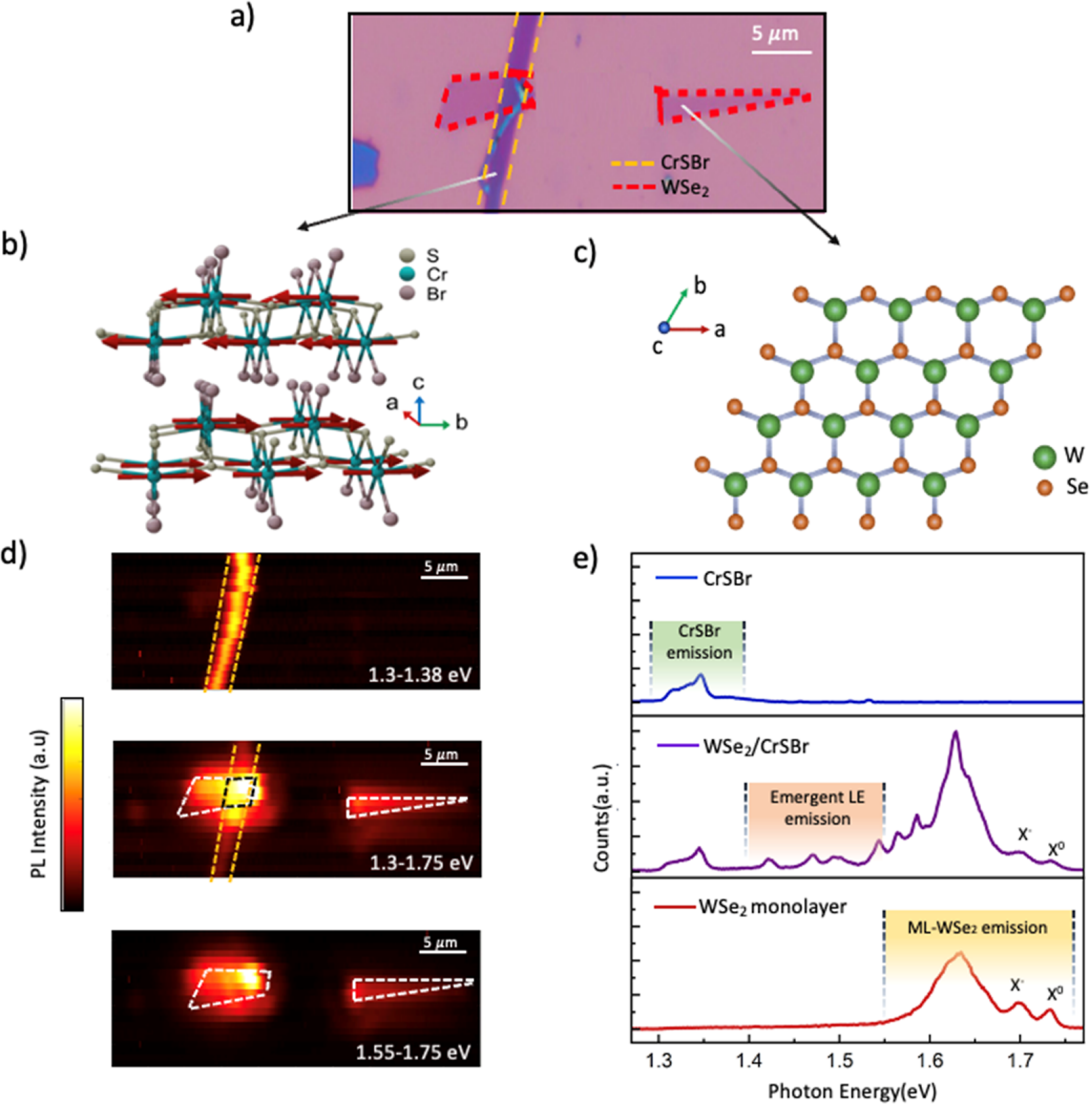
(a) Optical microscope image of the WSe_2_/CrSBr heterostructure over a silica on silicon substrate. The rectangular purple flake outlined by a yellow dashed line is a few-layer CrSBr flake (∼6 layers, determined by optical contrast), and the flake outlined by red dashed line is a monolayer of WSe_2_ placed on top of it. Note that the right WSe_2_ is separated and can be used as reference. (b,c) Crystal structure of the layered magnetic semiconductor CrSBr and monolayer WSe_2_, respectively. (d) Color map of the PL-integrated intensity of the sample at low temperature (∼30 K). The top and bottom panels represent the corresponding maps filtering just the contribution from CrSBr and WSe_2_, respectively, and the middle panel represents the map without any filtering. The reference layers (CrSBr flake and WSe_2_ monolayer) and the vdW heterostructure are marked by yellow, white, and black dashed lines, respectively. (e) Representative PL spectra from CrSBr reference (top), WSe_2_/CrSBr heterostructure (middle), and WSe_2_ reference (bottom), all at ∼30 K.

The relative intensities of the peaks in the heterostructure display notable differences in comparison to those in the WSe_2_ reference flake. First, the contributions of the neutral exciton and trion are clearly weaker than in the reference flake. Second, the emission of the heterostructure at energies typically ascribed to LEs presents a certain structure (i.e., clear contribution of isolated peaks), while that of the reference sample appears like a broad band. A more striking feature is the emergence of several emission lines at energies well below 1.6 eV. While the emission from LE typically occurs within the range of 1.6–1.75 eV,^5,17,18,41^ the emission of these lower energy peaks occurs in the range of 1.4–1.55 eV, suggesting higher localization potential. However, they must be tentatively ascribed to LEs in the WSe_2_ monolayer, as they exhibit valley Zeeman splitting under a perpendicular magnetic field (Figure S3).

Such low emission energy has been only found in interlayer and Moiré excitons in MoSe_2_/WSe_2_ heterostructures^23,44,45^ or monolayer WSe_2_ under strong strain fields.^46,47^ The first possibility can be disregarded due to the bandgap and crystalline structure of CrSBr. However, the strain origin is quite unlikely, although disputable, since such strong localization has not been observed on TMD monolayers suspended over any few-layer flake. After studying two more samples, we confirmed that it is indeed likely to find lower energy LEs in the CrSBr/WSe_2_ heterostructures. The different low energy peaks studied exhibited distinct behaviors, their emission energies being the only common parameter (see Figures S6–S9 for more details). Because of the variety of these peaks, they could not be ascribed to a specific excitonic specie. Given that TMDs are sensitive to their surroundings and because these emission energies have not been found in monolayer WSe_2_ suspended on other materials, we could assume that the adjacent CrSBr plays a role in the origin of these low-energy peaks.

For a better discussion about the influence of CrSBr on these peaks at lower energies, we focus our study on a bright LE occurring at 1.51 eV. The spectrum of this LE at 6 K is shown in Figure 2a shaded in light orange (see Figure S1D for the location). Alongside, we can clearly distinguish the emission from CrSBr and WSe_2_ (highlighted in light green and yellow, respectively). This new peak at 1.51 eV shows spectral jittering at low temperatures, a behavior commonly associated with highly LEs in 2D materials.^17^ Hence, a second-order photon-correlation measurement was carried out to confirm the single photon emission behavior (Figure 2b). The corresponding experimental data were fitted using the second-order photon-correlation function given by *g*(2)­(*t*) = 1 – (1 – *a*) exp­(−|*t*|/τ), where *t* is the delay time between simultaneous counts, τ includes pumping and recombination rates, and *a* gives the value of the function at *t* = 0. From these data, a *g*(2)­(0) < 0.5 is extracted, thus confirming a pronounced photon antibunching behavior characteristic of a SPE.^7–9^

**Figure 2:**
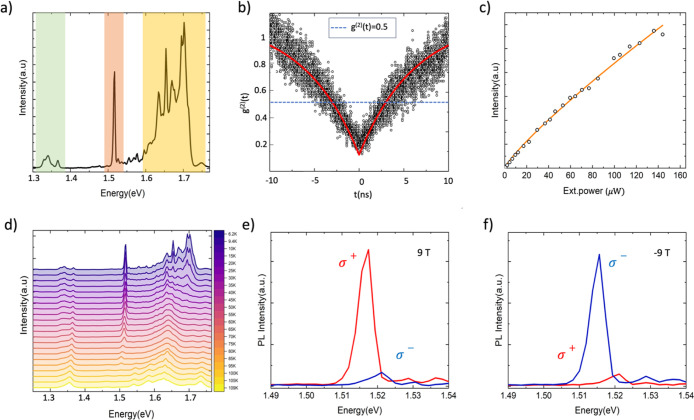
(a) μ-PL spectrum at ∼6 K from the region of the heterostructure. The shaded light green, orange, and yellow region represents the contribution to emission from few-layer CrSBr, the SPE (at 1.51 eV), and WSe_2_ monolayer, respectively. (b) Second-order photon correlation measurement of the SPE measured at 6 K. The scatter points represent the experimental data, and the red line is the fitted curve. (c) μ-PL intensity as a function of the incident laser power. (d) Temperature-dependent PL spectra from 4.5 to 250 K. (e,f) Circular polarization resolved spectra of the SPE for right (in red) and left (in blue) circular polarizations at different external perpendicular magnetic fields of +9 and −9 T, respectively.

The power dependence of the SPE shows a sublinear power dependence that substantiates the localized character of this emission (Figure 2c).^6,19,48^ A power exponent *a* ≈ 0.8 is obtained by fitting the data to the power law *I* = *P* ^*a*^. This value is in between the one expected for defect bound LEs (ca. 0.5)^49^ and strain-engineered emission (ca. 0.95).^50^ This deviation can be attributed to a rich doping environment combined with the interaction with defects from the magnetic substrate (the six-layer CrSBr in our case). As shown in Figure 2d, the SPE can emit up to relatively higher temperatures (100 K) (Figure 2d), suggesting a confinement potential higher than that usually observed in regular localization centers in WSe_2_ (see Figure S4).

Next, we studied the response of this emitter in the presence of an out-of-plane magnetic field. The corresponding polarization-resolved PL measurements at maximum negative and positive out-of-plane fields are shown in Figure 2e,f. We employed a combination of a quarter-wave plate and a linear polarizer at the detection to selectively measure the right- and left-circularly polarized components of the emission (Note that the excitation laser in unpolarized). The data reveal a highly polarized emission promoting σ+ or σ– circular polarized light depending on the direction of the magnetic field (Figure 2e,f). Importantly, the contribution from both valleys is equal in the absence of an external field (not shown). This result is very compelling as achieving highly polarized SPEs is often challenging without precise strain engineering, and even if so, polarization often depends on nature and orientation of strain wells.^17,18^ Only in very limited and specific conditions could the strain lead to equal σ+ and σ– contributions in the absence of an external magnetic field.

To conclude our experiments, in-plane magnetic field measurements were carried out to illustrate how the in-plane magnetic order of CrSBr influences the emission properties. The magnetic field dependence of the CrSBr flake is compared with the case of the SPE under study in Figure 3. It is well-known that few-layer samples of CrSBr undergo a spin flip transition around 200 mT in the presence of an in-plane magnetic field aligned along its easy axis.^51^ The signature of this transition in the PL is shown in the color plot in Figure 3a. Monitoring the SPE during the magnetic field switch, we can observe that this field-induced transition is closely correlated with a redshift in the energy of the SPE (Figure 3b). Note that this is observed just when the transition is getting started (with intermediate spin flips) around 200 mT and not when the complete transition occurs at 400 mT.^51^ This behavior is very different from the charge transfer reported by Serati de Brito et al. on the MoSe_2_/CrSBr heterostructure where the in-plane magnetic field only affects the relative peak intensities, without any energy shift.^24^

**Figure 3:**
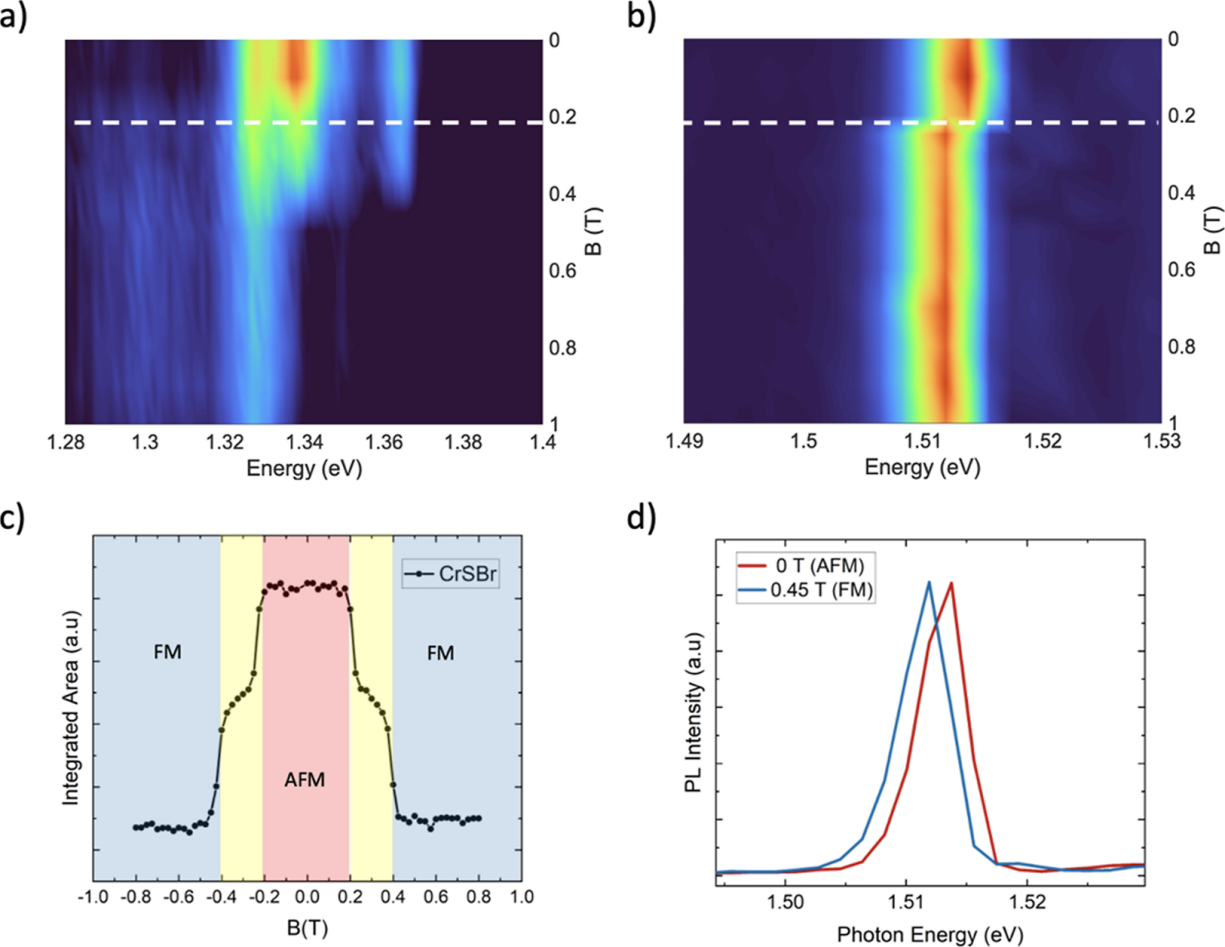
(a) The magnetic field response of CrSBr PL-integrated intensity for a field applied along the easy axis of CrSBr. The white dashed line marks the intermediate magnetic transition at around 0.2 T. (b) Same plot as (a) but for the SPE emission. The white dashed line marks the sharp shift at around 0.2 T. (c) The magnetic field dependence of the integrated area of the CrSBr PL band, the red, yellow, and blue region corresponds to the antiferromagnetic (AFM), intermediate, and ferromagnetic (FM) states of CrSBr. (d) Response of PL spectra of SPE to the magnetic transition of CrSBr induced by an in-plane magnetic field. The red and blue spectra indicate the SPE peak when the adjacent CrSBr layer is in AFM at 0 T and FM states at 0.45 T, respectively.

Comparing our results with current literature,^24,26,34,52^ we can mainly discuss about two key points that are of importance for valleytronic applications: (i) the correlation between the magnetic order of CrSBr and the emission of the SPE under study and (ii) the possible origin of the outstanding emission properties of this peak.

According to recent results, in type-II and type-III magnetic-semiconductor heterostructures, the magnetic order can induce spin-selective charge transfer, which may have an impact on the emission spectra of the semiconductor TMD constituent.^24,26,33^ However, any charge transfer in van der Waals heterostructures is mainly affecting the dynamics of the 2D excitons of the TMD monolayer, limited only by the thickness of the magnetic flake.^24,53^ For example, a clearly asymmetric magnetic coupling and magnetic order-dependent charge transfer has been recently reported in a heterostructure composed by monolayer MoSe_2_ on a bulk CrSBr.^24^ Even though it is measurable, the charge transfer observed in our sample was much lower due to a thinner CrSBr flake, as shown in the Supporting Information (see Section S5).

The existence of magnetic proximity effects could also explain the correlation between the emission and magnetic field. Despite the in-plane spin configuration of CrSBr, uncompensated magnetic moments or magnetic defects could cause an out-of-plane component when embedded in an antiferromagnetic material. However, if such a component exists, it might be weak as the WSe_2_ emission presents no insights into zero field splitting. However, we cannot definitively say that any zero-field splitting is absent as a wide range of zero-field splitting has been reported ranging from nonmeasurable splitting to up to 0.8 meV splitting.^6,54^

However, a simpler and more straightforward explanation would be related to the Coulomb interactions at the interface. The electronic band structure and the carrier mobility change in CrSBr during the transition from the antiparallel- to the parallel-spin configuration result in a different dielectric environment for the SPE depending on the spin configuration of CrSBr. It is well-known that the emission properties of the localization centers of TMDs are sensitive to the electronic environment. Attributing the energy shift of the SPE to these changes would explain the observed dependence on the in-plane magnetic field. In fact, deliberate changes in the environment are often employed for tuning the electronic band gaps and exciton binding energies,^55,56^ or for modifying the local Coulomb interactions by electrical gating.^57^

The discussion about the mechanisms correlating the emission and magnetism might as well be closely related to the very nature of this SPE. The local strain and impurities are the two common reasons for exciton localization in TMDs. Indeed, the emission of WSe_2_ can be shifted to 1.5 eV under uniaxial strain of the order of 2%.^58^ But notice that the neutral exciton disappears under such high strain fields. This is rather different to the situation shown in Figure 2, where an isolated peak at 1.51 eV coexists with the neutral exciton. In addition, just a good strain localization in WSe_2_ cannot adequately explain the in-plane magnetic field dependence of SPE. In this situation, the SPE can only be tentatively assigned to the interaction with defects located at the CrSBr flake. This hypothesis involves a sort of possibilities, including a valley-dependent electron–hole from WSe_2_ trapped by an intrinsic defect on CrSBr. For example, a negative trion bound to a magnetic defect could explain the sensitivity of our SPE to the in-plane magnetic field in contrast to the minor intensity change observed in the rest of the peaks. This could also be supported by the observation that the emergent LEs disappear abruptly around 40 K (Figure S4), coinciding with the hidden-order transition reported for CrSBr. The origin of this transition is still heavily debated, with some studies linking it to the ionization of donor impurities in CrSBr.^36,59,60^ Hence there appears to be a correlation between the defects in CrSBr and the emergence of low-energy LEs observed in our heterostructure.

In any case, we can conclude that independently of the nature of the SPE under study, the mechanisms correlating its optical and magnetic properties are bound to the presence of six-layer CrSBr. Quantitatively, the SPE sharply shifts around 2 meV, which is comparable to the Zeeman splitting energy observed for WSe_2_ emissions at a much higher out-of-plane field (9 T). That makes this kind of tuning appealing for low-operational-field applications. In addition, the in-plane magnetic field measurements also reveal a magnetic field dependence for the intensity of WSe_2_ main bands owing to charge transfer interaction (Figure S5), which could be more notable by replacing 6-layered CrSBr with a thicker CrSBr flake.

## Conclusion

We studied the magneto-optical properties of a van der Waals heterostructure consisting of a WSe_2_ monolayer interfaced with a few-layer CrSBr. Specifically, we have found several localized states emerging in WSe_2_ in the presence of adjacent CrSBr. Our attention is focused on a bright and highly polarized SPE operating at 1.51 eV, an emission energy only accessible by means of large strain fields for WSe_2_. Under an external out-of-plane magnetic field, this peak suffers regular Zeeman splitting, without any insight into spontaneous magnetization. However, in the presence of an in-plane magnetic field, the energy of the SPE is red-shifted approximately 2 meV. Importantly, this red-shift is clearly correlated with the metamagnetic transition of CrSBr, pointing out that its origin cannot be solely attributed to strain. Our approach enables contactless magnetic tuning of quantum light, which could be advantageous over conventional methods that rely on strain or electrical gating. Thus, given the relatively low magnetic field required for inducing the metamagnetic transition (on the order of 200 mT), leveraging in-plane magnetic ordering of CrSBr could enable rapid magnetic switching of highly polarized SPEs. Therefore, the proximity-type magnetic coupling in this heterostructure can open exciting possibilities for practical quantum information technologies and the development of functional quantum photonic devices.

## Methods

The thickness of the studied flakes was identified with an optical microscope by a calibrated contrast comparison method.^40^ μ-PL measurements were carried out on a μ-PL confocal system coupled to an attoDRY1000 cryostat and provided with a set of superconducting magnets. Inside the cryostat, the samples were placed on top of a piezoelectric three-axial stage for the accurate positioning of single flakes. A laser diode at a 520 nm wavelength with a power control driver was used as the excitation source. The diode emission was coupled to a monomode optical fiber to obtain a diffraction-limited spot with a power ranging from 0 to 100 μW. Then, backscattering was collected by means of a monomode optical fiber filtering the laser to obtain the μPL signal, which was analyzed with cooled silicon back-thinned CCD attached to a spectrometer.

## Supplementary Material



## Notes

Low-temperature PL spectra of the heterostructure; excitation power-dependent, out-of-plane magnetic field-dependent, temperature-dependent, and in-plane magnetic field-dependent PL data; and measurements from two additional samples (PDF)
