# Supplementary material for: Highly Polarized Single-Photon Emission from Localized Excitons in a WSe2/CrSBr Heterostructure
Source: ACS Photonics. 2025 May 29;12(6):3024–31. doi: 10.1021/acsphotonics.5c00144 (PMC12183727; doi:10.1021/acsphotonics.5c00144)
Supplement: Supplementary file 1 [file ph5c00144_si_001.pdf]

Supporting information for

Highly polarized single photon emission from localized excitons in a WSe_2/CrSBr heterostructure

Varghese Alapatt[1], Francisco Marques-Moros[1], Carla Boix-Constant[1], Samuel Mañas-Valero [1,2], Kirill I. Bolotin [3], Josep Canet-Ferrer[1], and Eugenio Coronado[1]**

[1]Instituto de Ciencia Molecular (ICMol), Universitat de València, c/Catedrático José Beltrán 2, 46980, Paterna, Spain

[2]Kavli Institute of Nanoscience, Delft University of Technology (TU Delft), Lorentzweg 1, 2628 CJ, Delft, The Netherlands

[3]Department of Physics, Freie Universität Berlin, Arnimallee 14, 14195, Berlin, Germany

Corresponding Authors: eugenio.coronado@uv.es, jose.canet-ferrer@uv.es

PL spectra of WSe_2/CrSBr heterostructure at 4K.

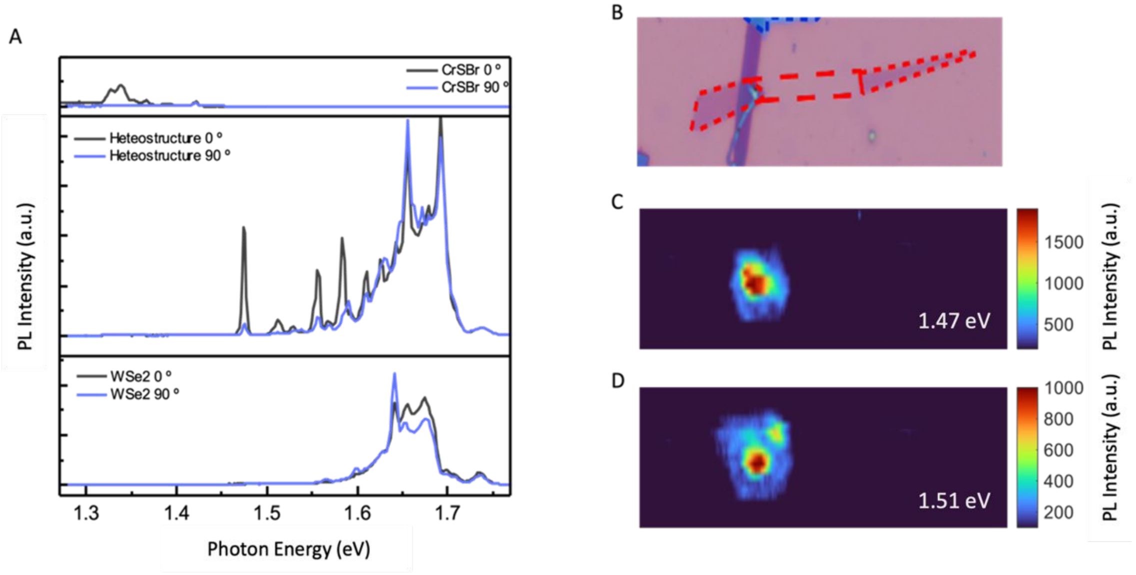

Figure S1. Representative PL spectrum of the WSe_2/CrSBr heterostructure acquired from the center of the heterostructure at 4K. (a) Linear polarization resolved PL from CrSBr reference (top), WSe_2/CrSBr heterostructure (middle), and WSe_2 reference (bottom), all at 4K. Note that the spectra from the heterostructure is from the same point as in Fig 1(c) of the main article. Black and blue spectra represent the linear polarization resolved PL parallel to and perpendicular to the easy axis of CrSBr respectively. (b) Optical microscope image of the heterostructure as described in the article. (c) and (d) The PL mapping at 1.47 eV and 1.51 eV.

We observe that the characteristic neutral exciton and trion of WSe_2 are not very prominent at very low temperature. The energy selected PL mappings show the localization of two emergent emissions at 1.47 eV and 1.51 eV. Note that the emission at 1.51 eV is the SPE described in the article, which appears highly localized at a point within the heterostructure.

Excitation power dependent PL of WSe_2/CrSBr heterostructure.

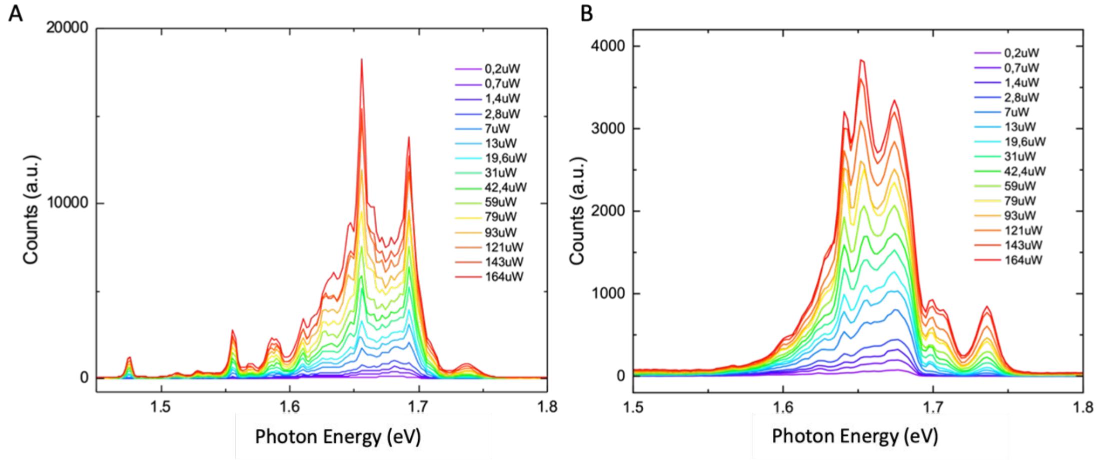

Figure S2. Excitation power dependent PL of the WSe_2/CrSBr heterostructure at 4K. (a) and (b) show the PL signals from the center of the heterostructure and the reference flakes at different excitation powers. These spectra are not resolved in polarization.

Out-of-plane magnetic field sweep.

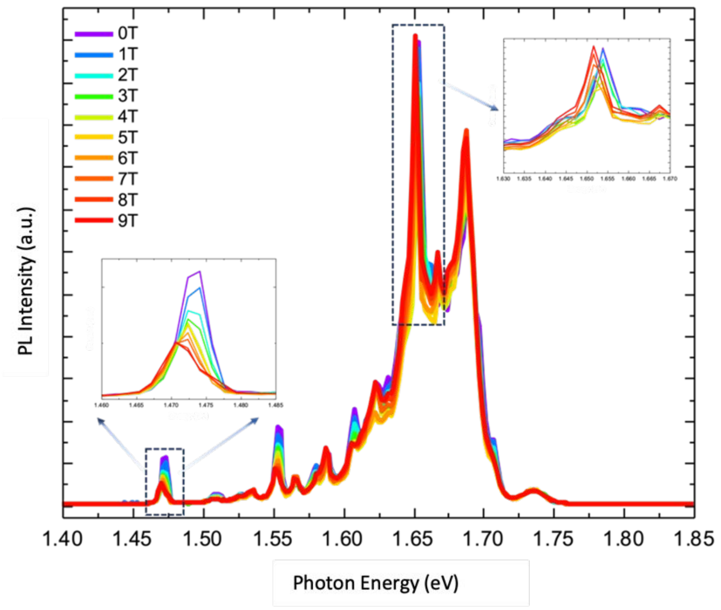

Figure S3. Photoluminescence dependence on the out-of-plane magnetic field from 0 to 9T. A redshift of around 2meV is observed coinciding with the extensively reported Zeeman spliting.[1] Plots for the isolated peak at 1.47 and narrow feature from the WSe_2 regular emission at 1.65eV are zoomed out as insets.

Temperature dependent PL of WSe_2/CrSBr heterostructure.

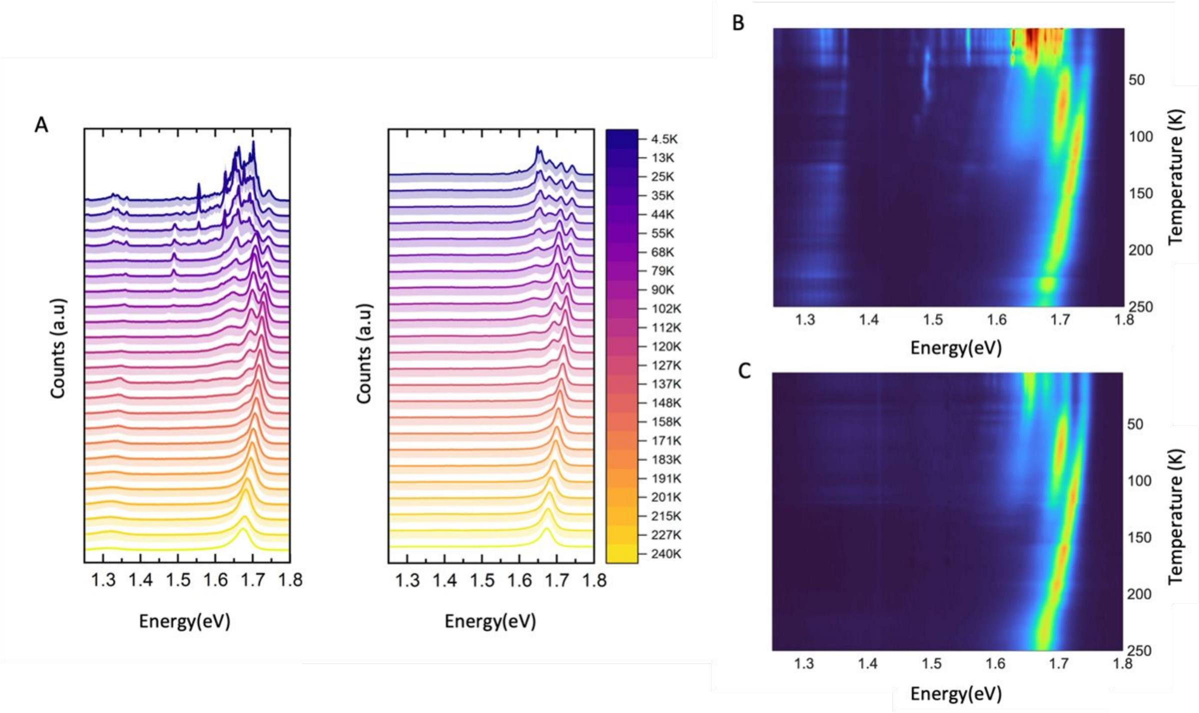

Figure S4. Photoluminescence dependence on the temperature. (a) Temperature-dependent PL spectra from the center of the heterostructure (left) and the reference WSe_2 monolayer (right) from temperatures from 4.5 to 250K. (b) and (c) Color map composed by the spectra from (a).

We observe that most spectral features in the heterostructure gradually disappear around 40K and the characteristic WSe_2 excitonic peaks recover their intensity and then follow the similar temperature dependence as in reference WSe_2.[1,2] Interestingly, the emergent emission from LEs are prominent below 40K which happens to coincide with the hidden-order transition temperature reported for CrSBr.[3,4]

In-plane magnetic field sweep

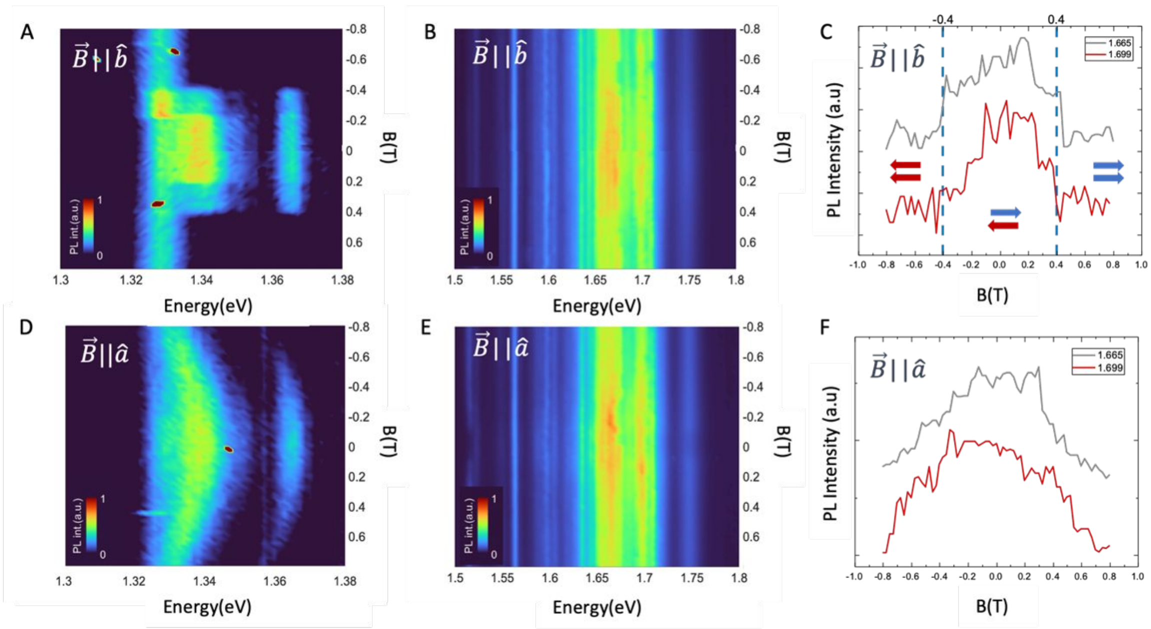

Figure S5. In-plane magnetic field dependent $\boldsymbol{\mu}$-PL measurements acquired at the center of the heterostructure. (a) and (b) Color plot showing the magnetic field dependence of the CrSBr and WSe_2 PL contributions (respectively) under an external in-plane magnetic field applied along the easy (b) axis of CrSBr. The color bar represents the intensity of the PL signal normalized to the corresponding maxima. The two contributions are plotted separately for better comparison as their intensities are very different. (c) The magnetic field dependence of the intensities of the two main peaks of WSe_2, at 1.665eV (grey) and 1.699eV (red) for field applied along the easy (b) axis of CrSBr. The dotted line is fixed at the flip field for the metamagnetic transition. (d) and (e) analogous color plots to (a) and (b), as a difference in this case external in-plane magnetic field is aligned in the intermediate (a) axis of CrSBr. (f) Analogous plot to (c) for the field oriented in the intermediate (a) axis of CrSBr.

This result is in agreement to the recently reported charge transfer interaction due to a broken type III band alignment in $MoSe_2$/CrSBr heterostructure.[5]

Peak statistics in additional heterostructures.

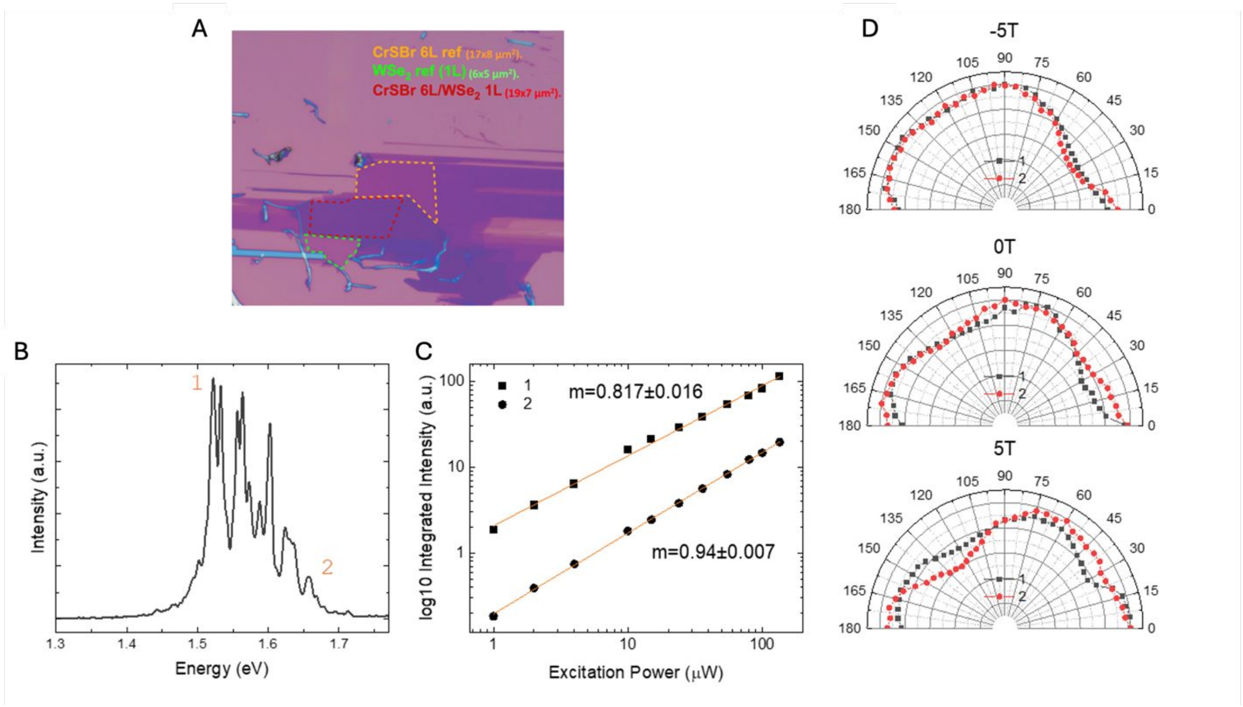

Figure S6 Sample II. (a) Optical image of the sample. The CrSBr, WSe_2 and heterostructure regions are marked by orange, green and red dashed-lines respectively. (b) Representative spectrum showing a set of emergent peaks emitting in the heterostructure below 1.6 eV. (c) Excitation power dependence of peaks 1 and 2 labelled in (b). (d) Circular polarization diagram of peak 1 and 2 with and without out-of-plane magnetic field. Note that the angles shown in the diagram correspond to the rotation angles of the quarter-wave plate (QWP). It has been fixed in such a way that the right- and left-circularly polarized (RCP and LCP) signals present a 90º phase delay. Hence within 0 and 180 degrees of rotation we can get both RCP and LCP contributions.

As shown in Figure S6 (b) and (c) a representative emergent peak at lower energy (peak 1 at around 1.51 eV, similar to the SPE in the article) and a representative peak from the WSe_2 emission range (peak 2) seems to have distinct excitation power dependence pointing to the distinct nature of the peaks. This once again, shows that the emergent peaks behave fundamentally different from the characteristic WSe_2 . Polarization dependence in Figure S6 (d) shows that there is no pronounced polarization selectivity in either of the peaks analysed here.

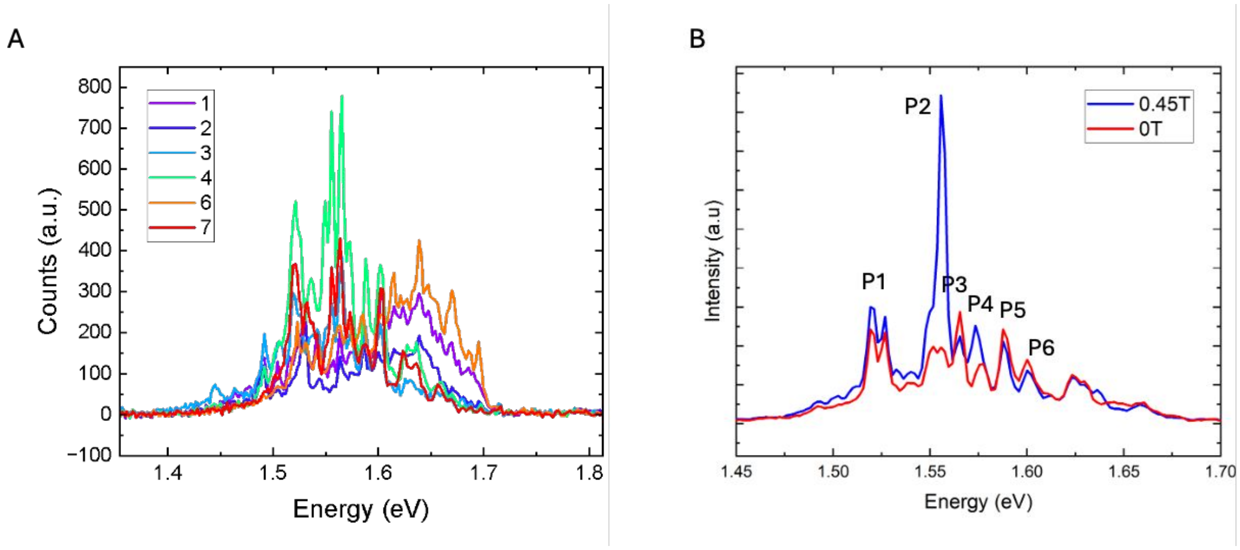

Figure S7 (a) PL spectra from seven different points on the heterostructure from heterostructure II. (b) The PL spectra at 0T (AFM CrSBr) and 0.45T (FM CrSBr) from the same point on the heterostructure for magnetic field applied along the easy axis of CrSBr.

As shown in Figure S7 (a), no clearly isolated emergent peaks are observed across the heterostructure. In Figure S7 (b), we see that different peaks exhibit distinct behaviours in response to the sharp spin-flip transition of CrSBr along its easy magnetization axis. Specifically, emissions P1, P3, and P6 show intensity variations, indicating charge transfer—an interaction already discussed in the Figure S5. Meanwhile, emissions P4 and P5 display energy shifts toward lower energies, similar to the SPE reported in the article; however, the magnitudes of these shifts differ (approximately 3 meV and 1.5 meV, respectively). Lastly, emission P2 undergoes significant changes in both intensity and spectral shape, making it challenging to analyse quantitatively.

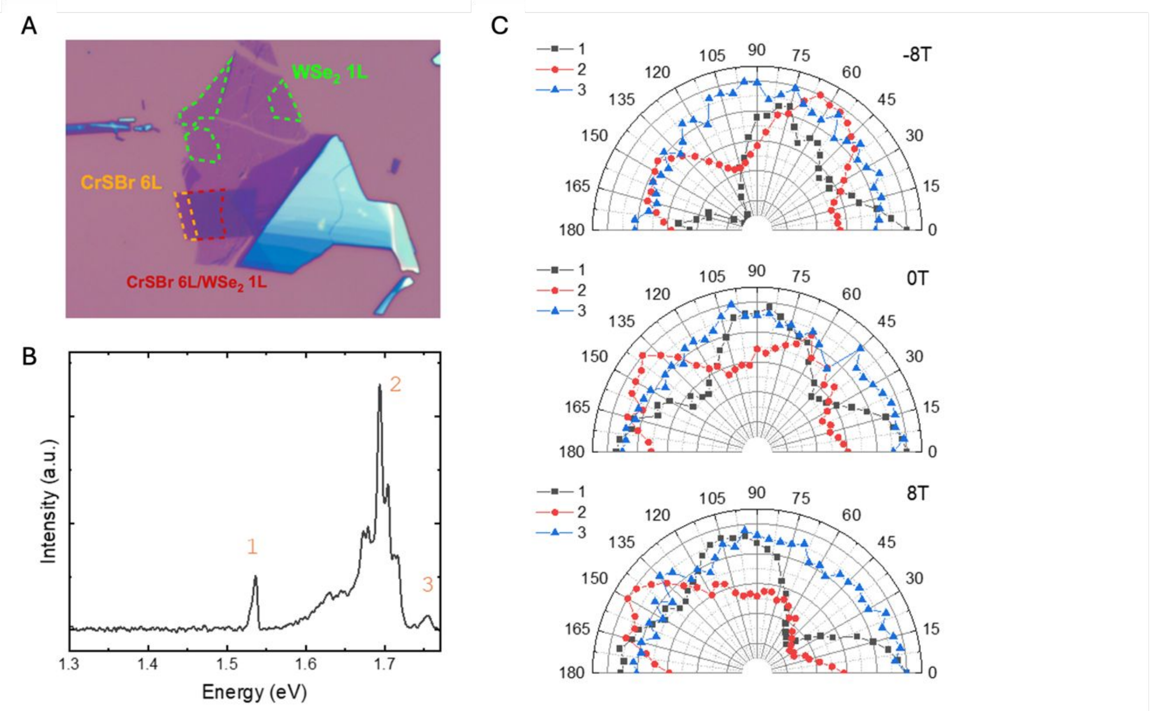

Figure S8. Sample III. (a) Optical image of the sample. The CrSBr, WSe_2 and heterostructure regions are marked by orange, green and red dashed-lines respectively. (b) Representative spectrum showing a single peak emitting below 1.6 eV. (c) Circular polarization diagram of Peak 1, 2, and 3 marked in (b) with and without out-of-plane magnetic field.

Unlike sample II, here we have a single isolated emergent peak at a very similar energy as that of the SPE in the article, however unlike the SPE this peak do not present any clear polarization selectivity with out-of-plane magnetic fields.

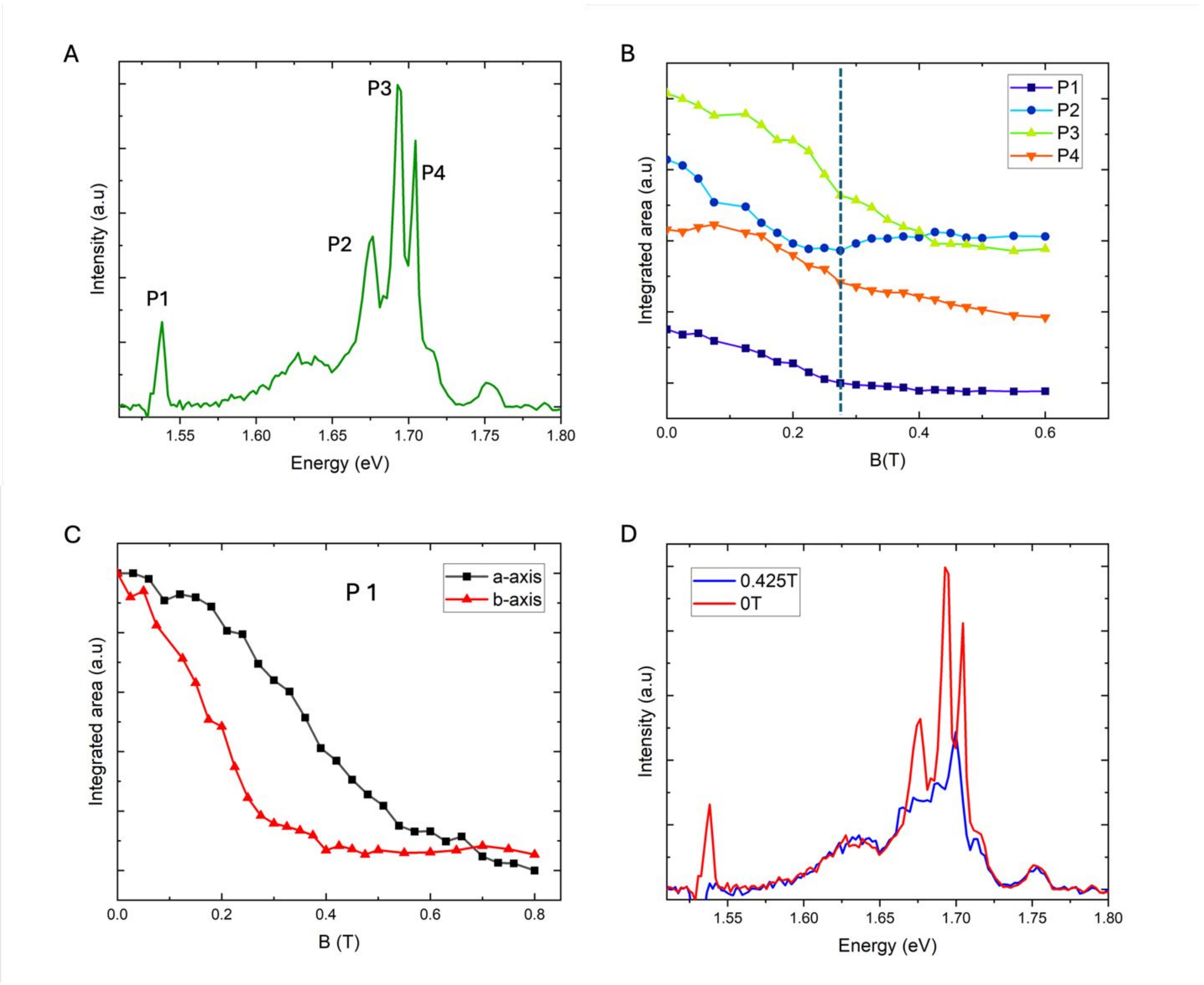

Figure S9. (a) PL signal from a point on the heterostructure from sample 3, where we observe a single isolated emergent peak at around 1.53 eV. (b) The response of the integrated areas of different emissions marked in (a) to the magnetic field applied along the easy axis of CrSBr. (c) The magnetic response of the integrated area of peak P1 for fields along the easy (red) and intermediate (black) axis of CrSBr. (d) The PL spectra at 0T (AFM CrSBr) and 0.45T (FM CrSBr) from the same point on the heterostructure for magnetic field applied along the easy axis of CrSBr.

Figure S9 presents the response of a single, isolated emergent emission from Sample 3 to in-plane magnetic fields. In this case, all emissions, including the emergent emission P1 at approximately 1.53 eV, exhibit strong involvement in charge transfer interactions. Notably, P1 nearly vanishes beyond the spin-flip transition in CrSBr, making it impossible to track any potential energy shift.

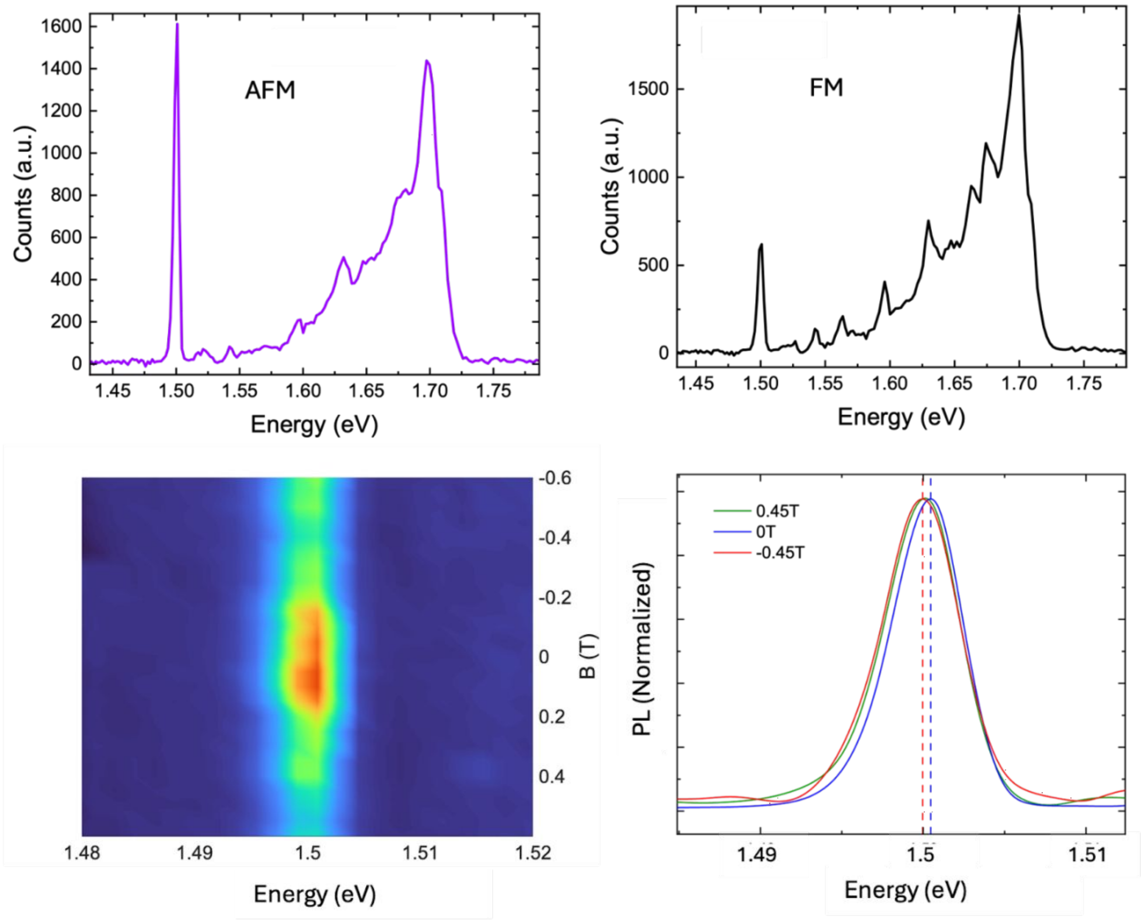

Figure S10. (a) and (b) PL signal from a point on the heterostructure, where we observe a single isolated emergent peak at around 1.5 eV in the AFM and FM configuration of underlying CrSBr, respectively (c) The magnetic field response of PL integrated intensity of this emission for field applied along the easy axis of CrSBr. (d) The PL spectra at 0T (AFM CrSBr), -0.45T (FM CrSBr) and 0.45T (FM CrSBr) from the same point on the heterostructure for magnetic field applied along the easy axis of CrSBr.

To further expand on this, we identified an emergent emission peak in one of the samples that closely matches the SPE in energy but exhibits a lower degree of localization (See Figure S10). Interestingly, this peak participates in magnetic-order-dependent charge transfer, unlike the SPE in the main text. At the same time, unlike the strongly quenched emission shown in Figure S9, this feature persists even after the antiferromagnetic-to-ferromagnetic (AFM-to-FM) transition, allowing us to directly track its spectral evolution. Upon normalizing the emission intensities in

the AFM and FM states, we observe a clear redshift and slight broadening in the FM phase. While the magnitude of the shift is smaller than that observed for the SPE, the spectral trend is qualitatively consistent.

REFERENCES

1. Huang, J.; Hoang, T. B.; Mikkelsen, M. H. Probing the origin of excitonic states in monolayer WSe2. *Scientific Reports* **2016**, *6* (1). https://doi.org/10.1038/srep22414.
2. Yan, T.; Qiao, X.; Liu, X.; Tan, P.; Zhang, X. Photoluminescence properties and exciton dynamics in monolayer WSe2. *Applied Physics Letters* **2014**, *105* (10). https://doi.org/10.1063/1.4895471.
3. Marques-Moros, F.; Boix-Constant, C.; Mañas-Valero, S.; Canet-Ferrer, J.; Coronado, E. Interplay between Optical Emission and Magnetism in the van der Waals Magnetic Semiconductor CrSBr in the Two-Dimensional Limit. *ACS Nano* **2023**, *17* (14), 13224–13231. https://doi.org/10.1021/acsnano.3c00375.
4. Boix-Constant, C.; Mañas-Valero, S.; Ruiz, A. M.; Rybakov, A.; Konieczny, K. A.; Pillet, S.; Baldoví, J. J.; Coronado, E. Probing the spin dimensionality in Single-Layer CRSBR Van der Waals heterostructures by Magneto-Transport measurements. *Advanced Materials* **2022**, *34* (41). https://doi.org/10.1002/adma.202204940.
5. De Brito, C. S.; Faria, P. E., Junior; Ghiasi, T. S.; Ingla-Aynés, J.; Rabahi, C. R.; Cavalini, C.; Dirnberger, F.; Mañas-Valero, S.; Watanabe, K.; Taniguchi, T.; Zollner, K.; Fabian, J.; Schüller, C.; Van Der Zant, H. S. J.; Gobato, Y. G. Charge transfer and asymmetric coupling of MOSE2 valleys to the magnetic order of CRSBR. *Nano Letters* **2023**, *23* (23), 11073–11081. https://doi.org/10.1021/acs.nanolett.3c03431.
